# Supplementary material for: Deficient uracil base excision repair leads to persistent dUMP in HIV proviruses during infection of monocytes and macrophages
Source: PLoS One. 2020 Jul 14;15(7):e0235012. doi: 10.1371/journal.pone.0235012 (PMC7360050; doi:10.1371/journal.pone.0235012)
Supplement: S1 Fig — The single nucleotide extension assay was used to establish the differences in dUTP/dTTP between MDM, MC and HAP1 dividing cells. The procedure is described in Methods. (a) Denaturing urea-PAGE of extension reactions in the presence and absence of dUTPase. The fold serial dilution of the dNTP extract used in each reaction is shown above each lane and establishes that the extension reaction is within the linear range of the assay (0.05–0.9 fraction substrate extended). The total [dTTP + dUTP] pool was 68 ± 6 pmol/million cells which was comprised entirely of dTTP (64 ± 4 pmol/million cells). (b) Single nucleotide extension reactions using cell extracts from monocytes (donor 2). The image is from one of two replicate measurements. Control reactions included polymerase in the absence and presence of added [dUTP + dNTPs] and dUTPase. (DOCX) [file pone.0235012.s002.docx]

**
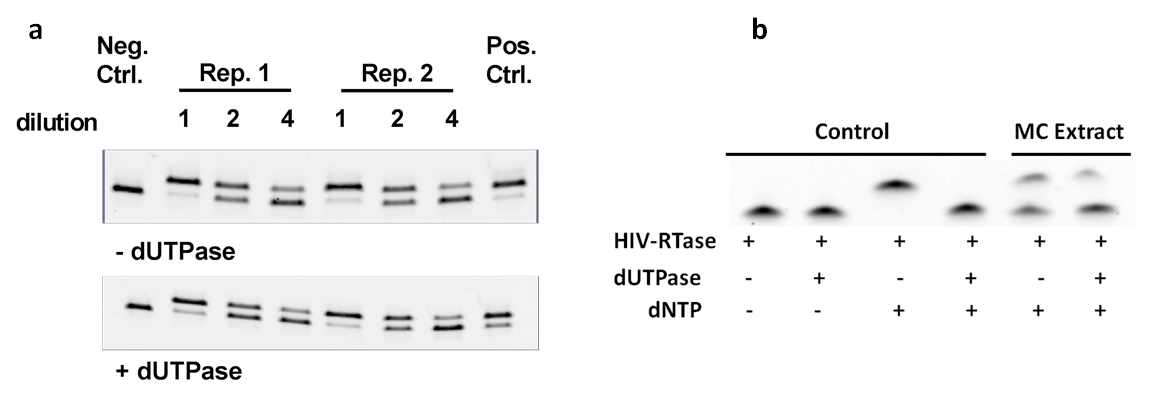
**

**S1 Fig. Measurement of dTTP and dUTP levels in the Hap1 dividing cell line.** The single nucleotide extension assay was used to establish the differences in dUTP/dTTP between MDM, MC and HAP1 dividing cells. The procedure is described in Methods. (**a**) Denaturing urea-PAGE of extension reactions in the presence and absence of dUTPase. The fold serial dilution of the dNTP extract used in each reaction is shown above each lane and establishes that the extension reaction is within the linear range of the assay (0.05-0.9 fraction substrate extended). The total [dTTP + dUTP] pool was 68 ± 6 pmol/million cells which was comprised entirely of dTTP (64 ± 4 pmol/million cells). (**b**) Single nucleotide extension reactions using cell extracts from monocytes (donor 2). The image is from one of two replicate measurements. Control reactions included polymerase in the absence and presence of added [dUTP + dNTPs] and dUTPase.
