# Supplementary material for: Deficient uracil base excision repair leads to persistent dUMP in HIV proviruses during infection of monocytes and macrophages
Source: PLoS One. 2020 Jul 14;15(7):e0235012. doi: 10.1371/journal.pone.0235012 (PMC7360050; doi:10.1371/journal.pone.0235012)
Supplement: S2 Fig — (a) Fluorometric activity assay for hUNG2 activity in Hap1 cell extracts (red data)(10 μg of total cell extract protein was used in the assay, see Methods). The a linear regression line through the data points is shown. The hUNG2 activity in Hap1 dividing cells is at least 25-fold greater than MDM and MC. For comparison, the black dashed line shows the equivalent activity present in MDM/MC as shown in Fig 2 of the main text. (b) mRNA expression levels of UBER enzymes in MDM relative to HAP1 dividing cells. Total RNA was extracted from MDMs after seven days differentiation from MC. The error bars in the qPCR measurements show the standard deviation from three replicate measurements. (DOCX) [file pone.0235012.s003.docx]

**
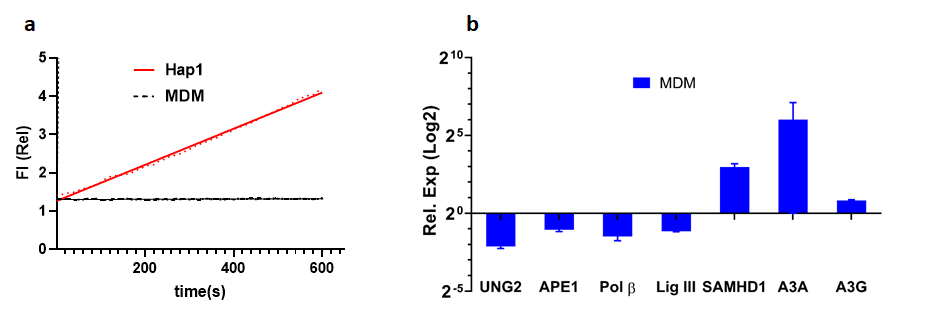
**

**S2 Fig.** **Determination of hUNG2 activity in extracts from Hap 1 cells and UBER mRNA expression levels in MDMs and comparison with the HAP1 dividing cell line. (a)** Fluorometric activity assay for hUNG2 activity in Hap1 cell extracts (red data)(10 μg of total cell extract protein was used in the assay, see Methods). The a linear regression line through the data points is shown. The hUNG2 activity in Hap1 dividing cells is at least 25-fold greater than MDM and MC. For comparison, the black dashed line shows the equivalent activity present in MDM/MC as shown in Figure 2 of the main text. **(b)** mRNA expression levels of UBER enzymes in MDM relative to HAP1 dividing cells. Total RNA was extracted from MDMs after seven days differentiation from MC. The error bars in the qPCR measurements are standard deviations from three replicate measurements.
