# Supplementary material for: Deficient uracil base excision repair leads to persistent dUMP in HIV proviruses during infection of monocytes and macrophages
Source: PLoS One. 2020 Jul 14;15(7):e0235012. doi: 10.1371/journal.pone.0235012 (PMC7360050; doi:10.1371/journal.pone.0235012)
Supplement: S4 Fig — (a) MC were infected with HIVNL4-3(eGFP) immediately after isolation at an MOI of ten. At 7-days post infection, eGFP expression was measured by flow cytometry. Even though GFP fluorescence is very low, viral reverse transcripts are abundant (main text). (b) Fully differentiated MDM were infected with HIVNL4-3(eGFP) at an MOI of ten. At 7-days post infection, eGFP expression was measured by flow cytometry. (DOCX) [file pone.0235012.s005.docx]

**
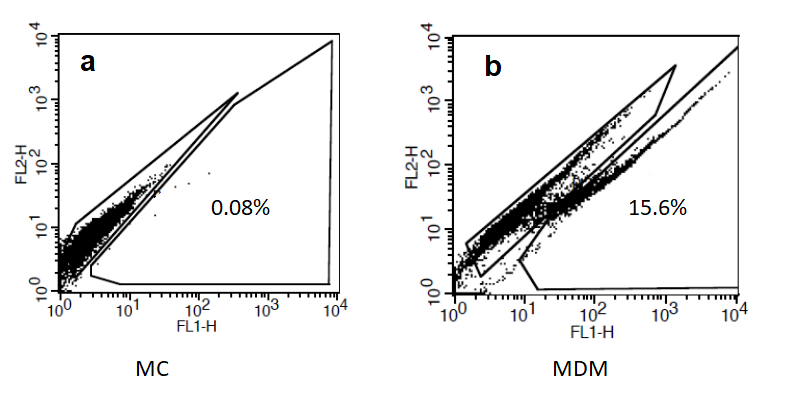
**

**S4 Fig. HIV-eGFP expression in MC and MDM.** (**a**) MC were infected with HIV^NL4-3(eGFP)^ immediately after isolation at an MOI of ten. At 7-days post infection, eGFP expression was measured by flow cytometry. Even though GFP fluorescence is very low, viral reverse transcripts are abundant (main text). (**b**) Fully differentiated MDM were infected with HIV^NL4-3(eGFP)^ at an MOI of ten. At 7-days post infection, eGFP expression was measured by flow cytometry.
