# Supplementary material for: Deficient uracil base excision repair leads to persistent dUMP in HIV proviruses during infection of monocytes and macrophages
Source: PLoS One. 2020 Jul 14;15(7):e0235012. doi: 10.1371/journal.pone.0235012 (PMC7360050; doi:10.1371/journal.pone.0235012)
Supplement: S6 Fig — Fully differentiated MDM were first transduced with inducible lentiviral construct expressing full length hUNG at an MOI of 5 (0.1 pg p24/cell) and 3 days later induced with doxycycline (1ug/ml). 1-day after induction, MDM were then infected with HIVNL4-3 single round virus at MOI of 0.5 (0.05 pg p24/cell). Total DNA was extracted at days 1, 3 and 7 and (a) LRT copies and (b) Frac U were measured by Ex-qPCR. (DOCX) [file pone.0235012.s007.docx]

**
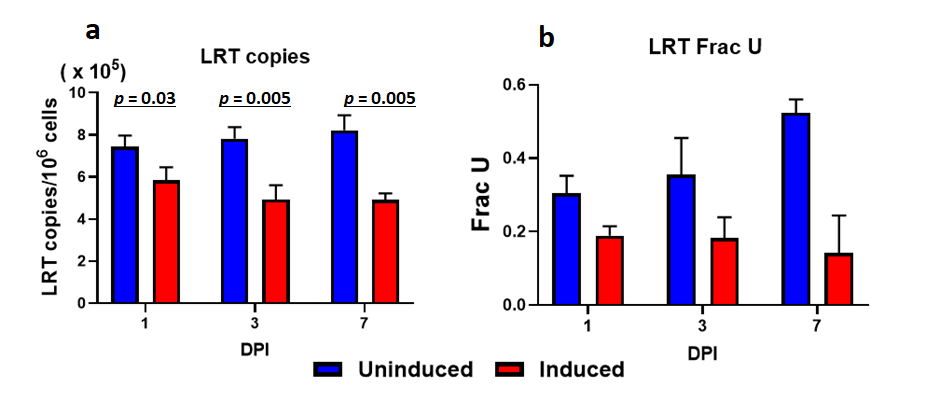
**

**S6 Fig. Effect of hUNG2 over expression in MDM on total HIV DNA copies.** Fully differentiated MDM were first transduced with inducible lentiviral construct expressing full length hUNG at MOI of 5 (0.1 pg p24/cell) and 3 days later induced with doxycycline (1ug/ml). 1-day after induction, MDM were then infected with HIV^NL4-3^ single round virus at MOI of 0.5 (0.05 pg p24/cell). Total DNA was extracted at days 1, 3 and 7 and (**a**) LRT copies and (**b**) The fraction of viral DNA containing uracil (Frac U) was measured by Ex-qPCR.
