# Supplementary material for: Deficient uracil base excision repair leads to persistent dUMP in HIV proviruses during infection of monocytes and macrophages
Source: PLoS One. 2020 Jul 14;15(7):e0235012. doi: 10.1371/journal.pone.0235012 (PMC7360050; doi:10.1371/journal.pone.0235012)
Supplement: S1 Raw Image — (DOCX) [file pone.0235012.s013.docx]

**Supplemental Figures**

**Figure S6. Fig 1_Raw_Image**

**Figure S7. Fig S1_Raw_Image**

**Figure S8. Fig 2c and 2d_Raw_Image**

**Figure S9. Fig 2e and 2f_Raw_Image**

**Figure S10. Fig 2g and 2h_Raw_Image**

**Figure S11. Fig 2I and 2j_Raw_Image**

**Figure S12. Fig S5_Raw_Image**

**
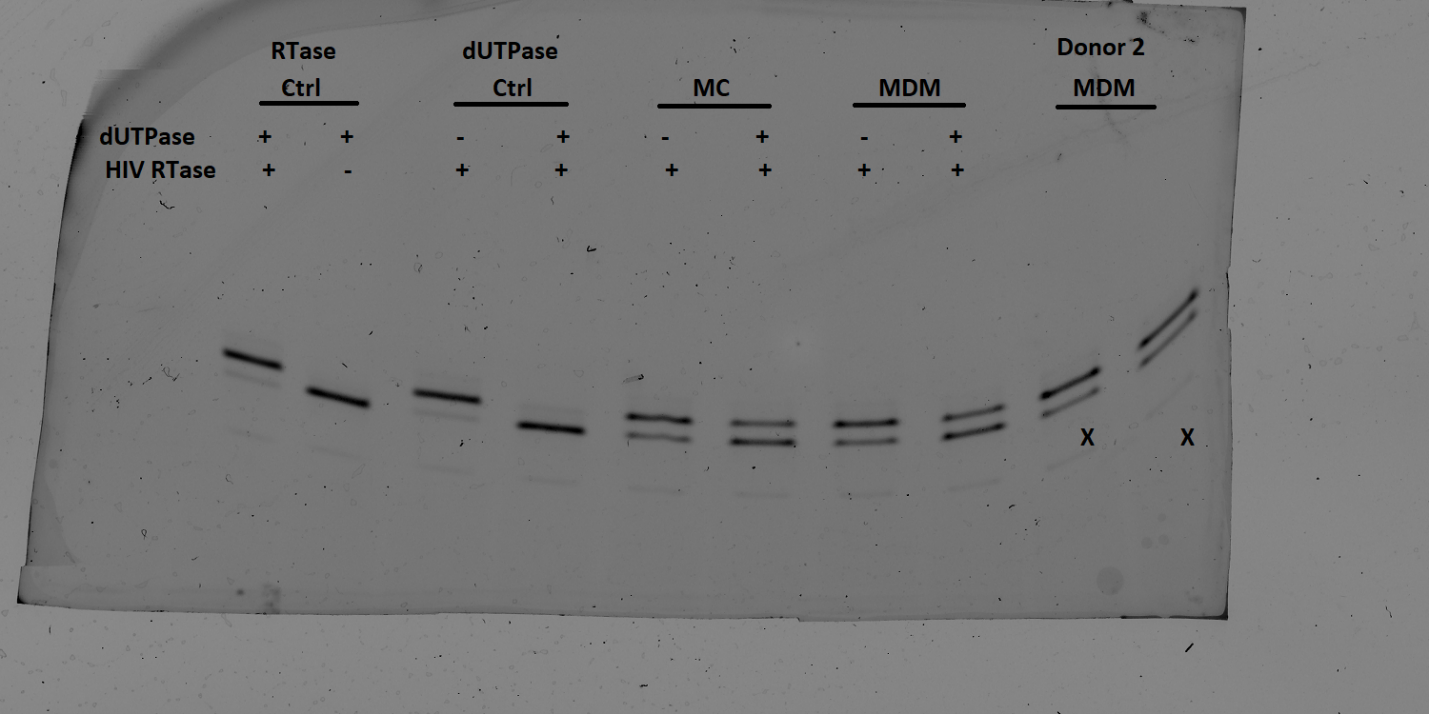
**

**Figure S6. Fig 1_Raw_Image.** Single nucleotide assay to detect dUTP and dTTP levels in MC and MDM. Image was captured on Typhoon fluorescence imager at 800 V. Donor two is not included in Figure 1. The rest are labeled as they appear in Figure 1.


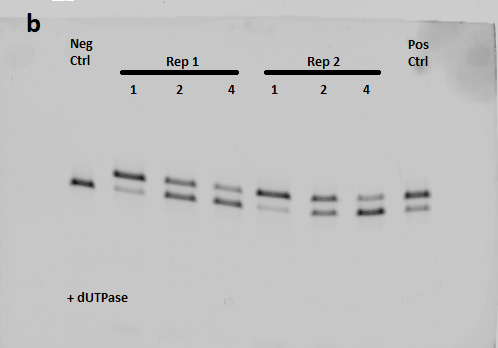

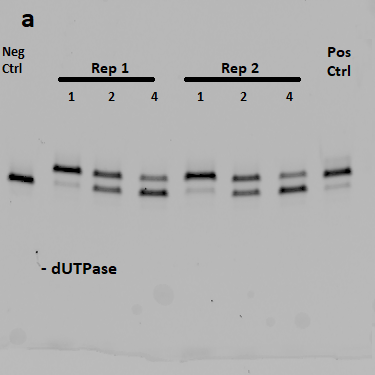


**
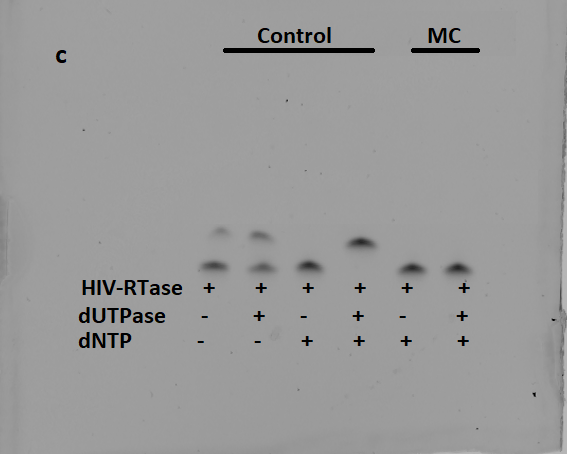
**

**Figure S7. Fig S1_Raw_Image** Single nucleotide extension assay. Images were captured on a Typhoon fluorescence imager at 650 V. The images in panels (a) and (b) correspond to Fig S1a and panel (c) corresponds to Fig S1b.

**
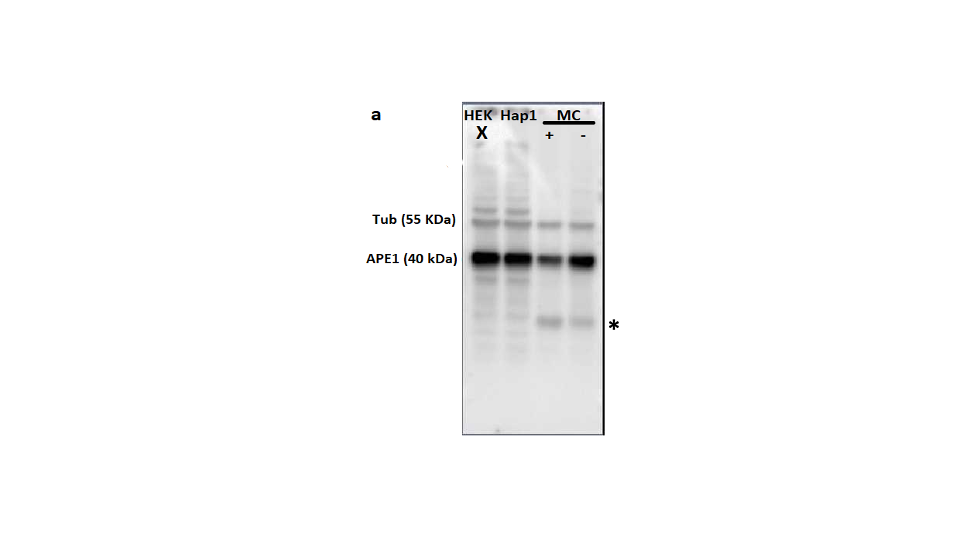

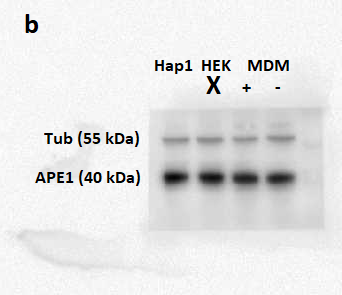
**

**Figure S8**. Fig 2c and 2d_Raw_Image. APE1 western blot images using MC (panel a, Fig 2c) and MDM (panel b, Fig 2d) protein extracts. The image was captured on a GelDoc imager (BioRad) using a 15 second exposure. The asterisk marks an unknown low molecular weight protein band.


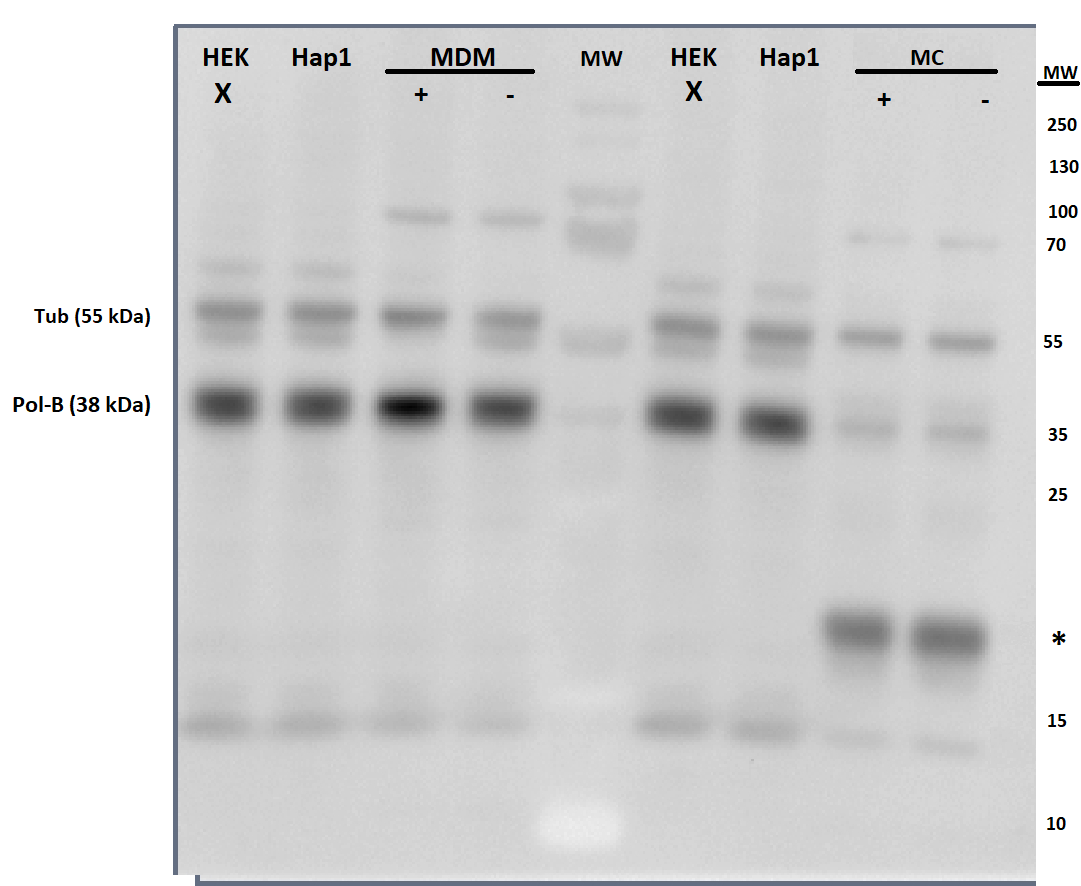


**Figure S9**. Fig 2e and 2f_Raw_Image. Western blot image for pol β using protein extracts from MC (2e) and MDM (2f). Image was captured on GelDoc imager (BioRad) using a 15 second exposure. The asterisk marks an unknown low molecular weight protein band.


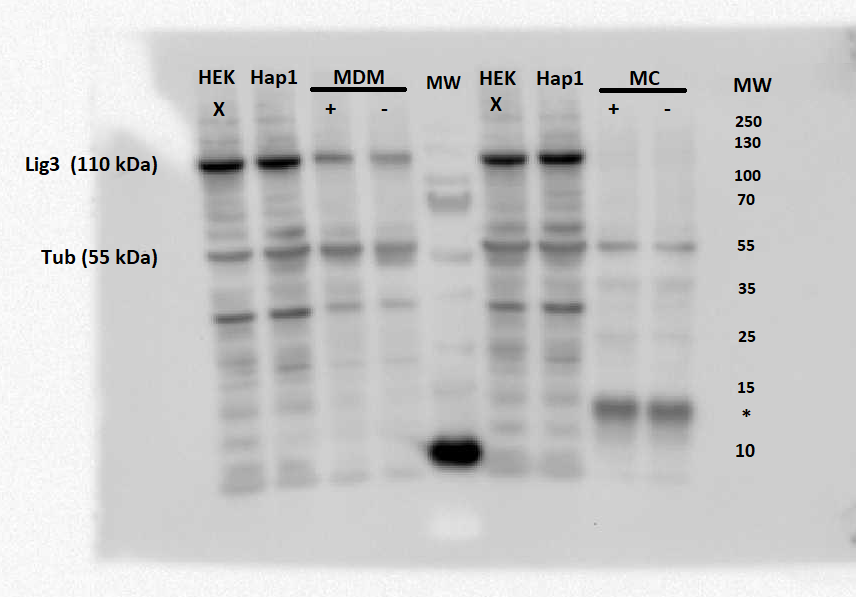


**Figure S10**. Fig 2g and 2h_Raw_Image. Lig3α western blot image from protein extracts of MC (2g) and MDM (2h). Image was captured on a GelDoc imager (BioRad) using a 15 second exposure. The asterisk marks a nonspecific band that is stained by the tubulin antibody in heavily developed images.


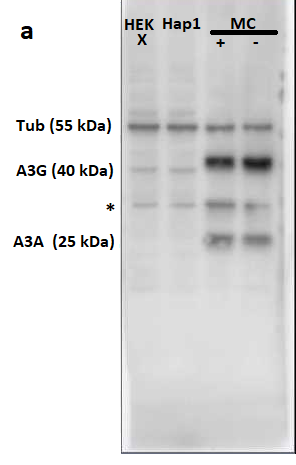

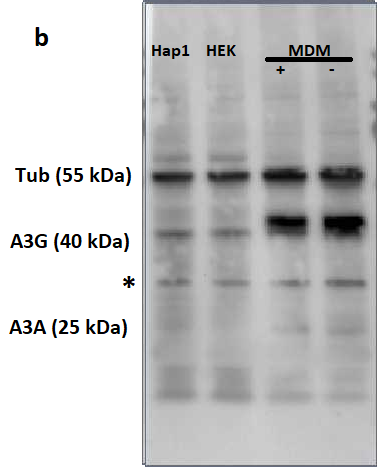


**Figure S11**. Fig 2i (a) and 2j (b)Raw_Images. A3G and A3A western blot image using MC (panel a, Fig 2i) and MDM (Panel b, Fig 2j) protein extracts. The image was captured on a GelDoc imager (BioRad) using a 15 second exposure. The asterisk marks a nonspecific band that is stained by the tubulin antibody in heavily developed images.


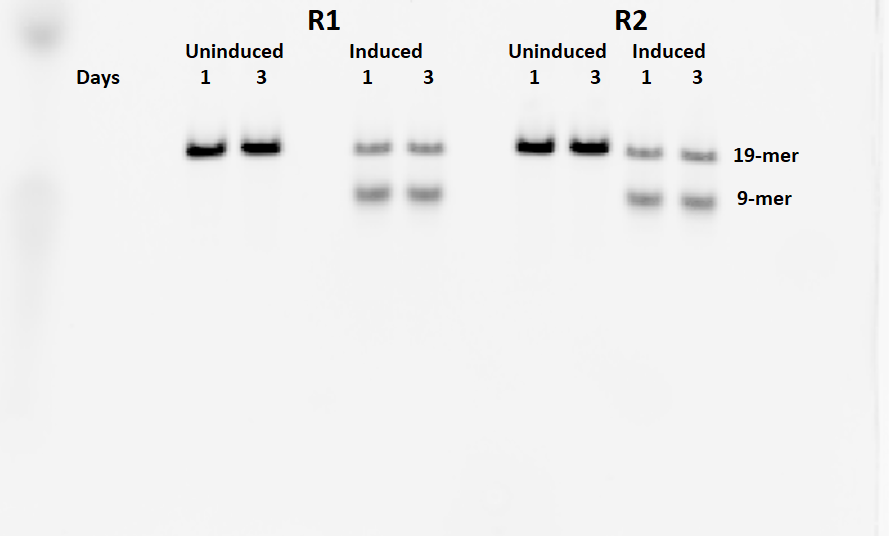


**Figure S12**. FigS5_Raw_Image. Fluorescence image was captured on Typhoon imager at 400 V. R1 and R2 are replicate measurements.
